# Activation of NRF2 by dexamethasone in ataxia telangiectasia cells involves KEAP1 inhibition but not the inhibition of p38

**DOI:** 10.1371/journal.pone.0216668

**Published:** 2019-05-20

**Authors:** Sara Biagiotti, Marzia Bianchi, Luigia Rossi, Luciana Chessa, Mauro Magnani

**Affiliations:** 1 Department of Biomolecular Sciences, University of Urbino, Urbino, Italy; 2 Department of Clinical and Molecular Medicine, Sapienza University of Rome, Rome, Italy; University of PECS Medical School, HUNGARY

## Abstract

Oxidative stress has been shown to play a crucial role in the pathophysiology of the neurodegenerative disease Ataxia Telangiectasia. We have recently demonstrated that Dexamethasone treatment is able to counteract the oxidative state by promoting nuclear factor erythroid 2-related factor 2 (NRF2) nuclear accumulation. However, substantial gaps remain in our knowledge of the underlying molecular mechanism(s) according to which Dexamethasone acts as an NRF2 inducer. Herein we investigate the possible effects of the drug on the main NRF2 activation pathways by initially focusing on key kinases known to differently affect NRF2 activation. Neither AKT nor ERK1/2, known to be NRF2-activating kinases, were found to be activated upon Dexamethasone treatment, thus excluding their involvement in the transcription factor nuclear shift. Likewise, GSK3 inactivating kinase was not inhibited, thus ruling out its role in NRF2 activation. On the other hand, p38 MAPK, another NRF2-inhibitory kinase, was indeed switched-off in Ataxia Telangiectasia cells by Dexamethasone-mediated induction of DUSP1 phosphatase, and therefore it appeared that it might account for NRF2 triggering. However, this mechanism was excluded by the use of a selective p38 inhibitor, which failed to cause a significant NRF2 nuclear shift and target gene induction. Finally, dexamethasone effects on the classical oxidative pathway orchestrated by KEAP1 were addressed. Dexamethasone was found to decrease the expression of the inhibitor KEAP1 at both mRNA and protein levels and to induce the shift from the reduced to the oxidized form of KEAP1, thus favouring NRF2 translocation into the nucleus. Furthermore, preliminary data revealed very low levels of the negative regulator Fyn in Ataxia Telangiectasia cells, which might account for the prolonged NRF2-activated gene expression.

## Introduction

Ataxia Telangiectasia (AT) is a rare, incurable, neurodegenerative disease caused by biallelic mutations in the ATM gene [1, 2], which code for ATM, a protein of the PI3K family [3]. The lack of this single kinase leads to a highly pleiotropic phenotype [4–8]. The pathophysiological process that underlies the disease is not completely understood, but emerging evidence suggests that oxidative stress plays a crucial role [9–13]. In the last few years, steroids have been studied extensively because they have been shown to attenuate the neurological symptoms of the disease [14–20]. Indeed, experimental studies have been conducted to identify the molecular mechanism(s) underlying their efficacy [21–28].

In our laboratory, we have discovered a new ATM transcript induced by Dexamethasone (DEX). This transcript can prompt the translation of a short ATM variant with residual kinase activity, suggesting that drug treatment can provide some gain of functions by directly replacing the kinase that is lacking [22, 28]. In addition, we have identified several pathways, enhanced by DEX at least at the transcriptional level, which can work together with the “miniATM” isoform to activate different cellular responses and partially compensate for full ATM functions [24, 27]. Recently, we have observed that DEX is able to significantly increase the levels of the endogenous antioxidants GSH and NADPH [25]. With respect to the molecular mechanisms, we have found that most of the antioxidant enzymes involved in GSH homeostasis and NADPH production are upregulated. Interestingly, we considered that the expression of all these enzymes could be under the control of NRF2, the master regulator of the antioxidant response [29]. To prove this hypothesis we showed that DEX promotes the nuclear shift of NRF2, thus activating the transcription of these antioxidant genes and, ultimately, improving the redox state of AT cells [25]. To the best of our knowledge, this was the first investigation suggesting that a glucocorticoid analogue could act as an NRF2 inducer. Due to the novelty of the finding, the mechanism of NRF2 activation by DEX is still unknown. On the other hand, there is growing interest in NRF2 as a therapeutic target in the treatment of neurodegenerative diseases [30, 31]. Thus, unravelling the molecular mechanism(s) of DEX-induced NRF2 activation in AT cells could provide new insights into the role of glucocorticoids in AT and other neurodegenerative diseases.

Hence, the aim of the present investigation was to determine how DEX exerts its effect on the NRF2-ARE system, focusing on the main pathways that are known to be involved in NRF2 activation. First, we investigated the effect of DEX on the PI3K/AKT and ERK1/2 pathways, excluding the involvement of these activating kinases. We subsequently investigated the effects of DEX on the inhibitory kinases GSK3 and p38 MAPK, finding evidence that p38 inhibition by DEX could be involved. However, experiments with p38 inhibitors suggested that p38 inhibition may not account for full NRF2 activation. Hence, we refocused our investigation on the oxidative branch of NRF2 regulation, via the KEAP1/NRF2 axis. Surprisingly, it was found that the greatest effects of DEX could be accounted for by direct inhibition of KEAP1 at both transcriptional and post-translational levels. In short, we showed that DEX decreased both KEAP1 gene transcription and protein expression and that, interestingly, it was able to induce the shift from the reduced to the oxidized form of KEAP1, thus favouring NRF2 translocation into the nucleus.

## Materials and methods

### Cell lines

EBV-transformed lymphoblastoid B cell lines (LCLs) established from AT patients (ATM-/-) and healthy donors (ATM+/+) were kept in RPMI1640 medium supplemented with 2 mmol/l L-glutamine, 50 mg/ml gentamycin, and 10% fetal calf serum. The LCL AT cell lines AT28RM, AT50RM and AT129RM were already described in [25]. All cells were incubated at 37°C with 5% CO_2_ and treated with 100 nM DEX for 24h prior to RNA and protein extractions. Dimethylsulfoxide (DMSO) was used as the drug vehicle and thus administered in untreated cells as a control. All the cells used in this study were established for diagnostic purposes under informed consent and stored at the Department of Clinical and Molecular Medicine, Sapienza University of Rome. The cell lines were anonymized and made available only by a coding number, so that we had no access to the name of the patients. All the cell lines were used with the approval of the ethical committee of the Sant’Andrea Hospital.

### Quantitative Real-Time PCR

Total RNA was extracted from LCLs treated or not treated with DEX using the RNeasyPlus mini kit (Qiagen), and cDNA was obtained by PowerScript reverse transcriptase (Clontech). Quantitative PCRs for gene expression were performed by TaqMan Gene Expression Assays using the TaqMan Gene Expression Master Mix (Life Technologies) and run on a 7500 Real-Time PCR System (Applied Biosystems). HPRT1 was used as a housekeeping gene, as specified.

### Western blotting

Western blots were performed on WT and AT cell protein extracts, essentially as described in [25]. After 24-h treatment, cells were collected, washed, and lysed in denaturing lysis buffer for total protein extracts. For cytosolic/nuclear protein extracts, cells were subjected to hypertonic lysis to obtain the cytosolic fraction and subsequent denaturing lysis on the nuclear pellet. Protein extracts were quantified, loaded onto the SDS-PAGE, transferred onto the nitrocellulose membrane and then subjected to immunoblotting. The following antibodies from Cell Signalling Technologies were used in the immunoblotting analyses: Phospho-AKT (Ser473), AKT, Phospho-p44/42 MAPK (ERK1/2) (Thr202/Tyr204), p44/42 MAPK (ERK1/2), Phospho-GSK-3α/β (Ser21/9), GSK-3α/β, Fyn, LAMIN A/C, p38 MAPK, Phospho-p38 MAPK (Thr180/Tyr182) and KEAP1. Anti-MKP1, NRF2, HPRT1 and β-ACTIN were obtained from Santa Cruz Biotechnology as reported in [25]. Imaging and quantification were performed using a ChemiDoc MP Analyzer and image lab software (Bio-rad).

### Redox western

Protein extracts for redox western were obtained in non-reducing lysis buffer, essentially as described in [32], but on fractionated protein extracts. Cells were washed in phosphate buffer saline containing 40 mM NEM and kept on ice during the whole extraction. Lysis was obtained by hypotonic lysis buffer (10 mM HEPES, pH 7.8, 10 mM KCl, 2 mM MgCl_2_, 0.1 mM EDTA, 0.2 mM NaF, 0.2 mM Na_3_VO_4_) with freshly added protease inhibitors and 40 mM NEM. The suspensions were incubated on ice for 5 min, and Nonidet P40 was added to a final concentration of 0.6%. Following centrifugation at 16000 g, the pellet (nuclei) and the supernatant (cytosol) were separated. Nuclear extracts were then obtained by mild sonication in NEM lysis buffer. Both the cytosolic and nuclear extracts were diluted in 4X Sample buffer and analysed by Western blot or Redox Western blot, as described below. Half of the sample was reduced by the addition of 200 mM DTT and loaded on 4–15% SDS-PAGE to detect reduced forms of KEAP1, while the remaining extracts were loaded on a separate 4–15% SDS-PAGE under nonreducing conditions to evaluate oxidized forms of KEAP1.

### Immunoprecipitation

For Immunoprecipitation (IP), cytosolic and nuclear extracts were incubated with NRF2 rabbit polyclonal antibody (Santa Cruz Biotechnology), and the immune complexes were pulled-down by incubation with protein A Sepharose Fast Flow Beads (GE Healthcare). Briefly, equal amounts of cytosolic and nuclear extracts belonging to the different samples (40 micrograms of total proteins) were first denatured with 1% SDS to disrupt association of NRF2 with specific interactors. They were then subjected to IP protocol before being electrophoresed on SDS-PAGE and transferred to nitrocellulose membranes. Immunoblotting was performed with Phospho-Tyrosine Mouse mAb (Cell Signaling Technologies) or anti-Ub mouse monoclonal antibody (Santa Cruz Biotechnology) for phosphorylation and ubiquitination status, respectively.

### Statistics

Statistical analyses were performed with GraphPad Prism 5 software for Windows using the paired non-parametric Wilcoxon signed-rank test. P-values < 0.05 were considered significantly different.

## Results and discussion

**Fig 1:**
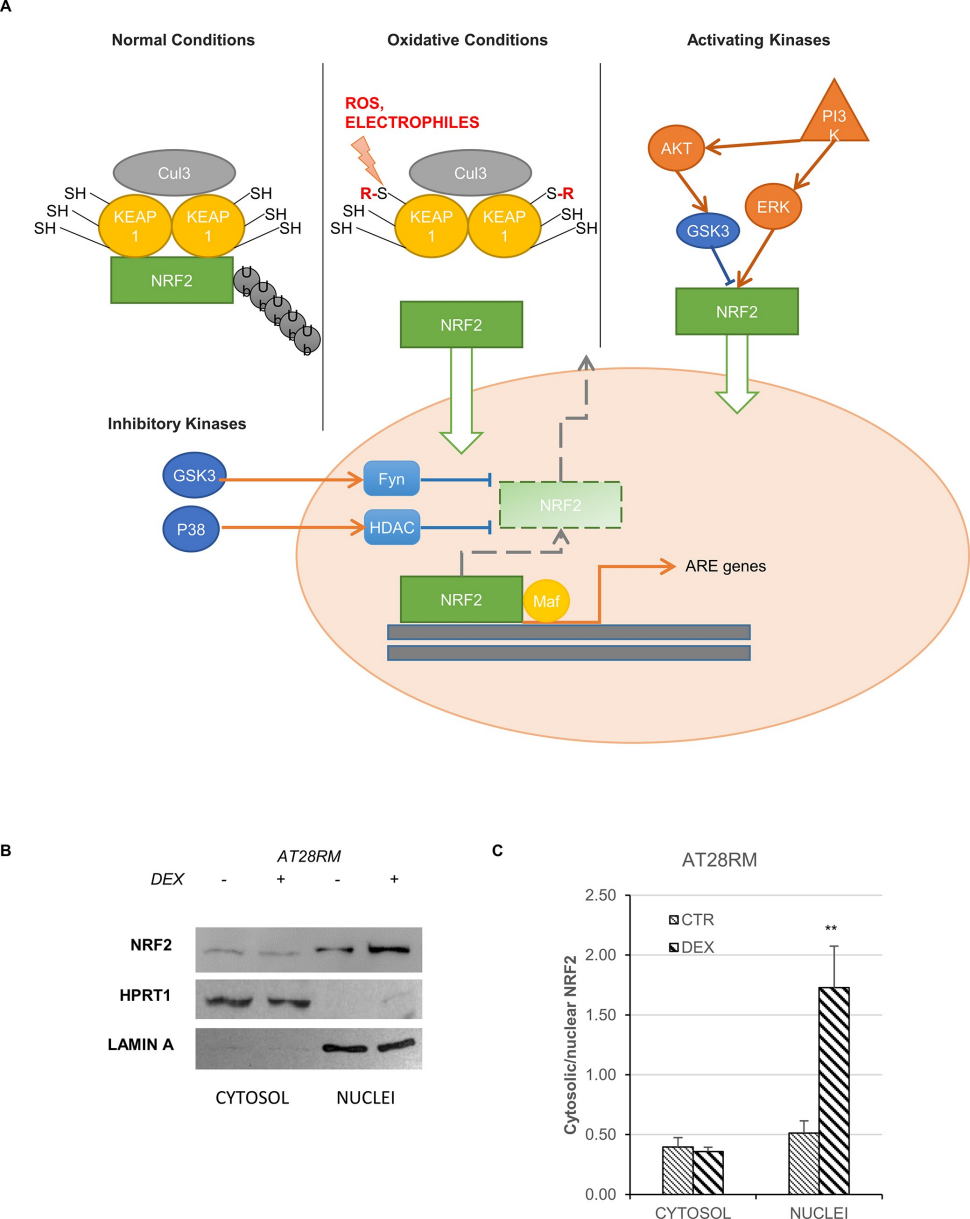
NRF2 activation in healthy and diseased conditions (modified from [31]) and the effect of DEX. **A)** Under normal conditions NRF2 is kept at low steady state levels in the cytoplasm, a process which is tightly regulated by interaction with the Cul3 protein-adaptor KEAP1 (first branch). Under oxidative conditions, KEAP1 acts as a redox sensor releasing NRF2 and allowing its nuclear shift and the expression of target genes (second branch). In addition to the known ways of activation, phase II antioxidant enzyme induction can also be triggered by activating kinases, allowing NRF2 nuclear transport in a KEAP1-independent manner. This involves PI3K/AKT activation and/or GSK3-dependent modulation (third branch). Lastly, under chronic conditions, such as neurodegenerative diseases, the constitutive activation of inhibitory kinases leads to NRF2 detachment from ARE genes, inhibiting the antioxidant response (fourth branch). **B),C)** NRF2 nuclear translocation in the AT28RM cell line upon DEX treatment. **B)** Western blot analysis for NRF2 in the cytosolic/nuclear fractions of AT28RM cells treated with 100 nM DEX for 24 hours or not treated. HPRT1 and LAMIN A served as a loading control for the cytosolic/nuclear extracts, respectively. **C)** Quantification of the relative amounts of NRF2 in the cytosol and nuclei of AT28RM cells. Blot shown is representative and the histograms are the means and SEM of four independent experiments. (Wilcoxon signed rand test; *two-tailed p-values<0.05).

NRF2 could be activated by different pathways, which are summarized in **Fig 1A**. Moreover, we recently published, and herein confirmed, that DEX is able to strongly promote the nuclear translocation of NRF2, especially in AT cells [24], (**Fig 1, panels B, C**). Our initial approach to investigate the molecular pathway(s) underlying the DEX effect was to focus on three different pathways for NRF2 activation. These pathways are thoroughly reviewed in [30, 33, 34] and depicted in **Fig 1A**.

### Effect of DEX on NRF2-activating kinases

**Fig 2:**
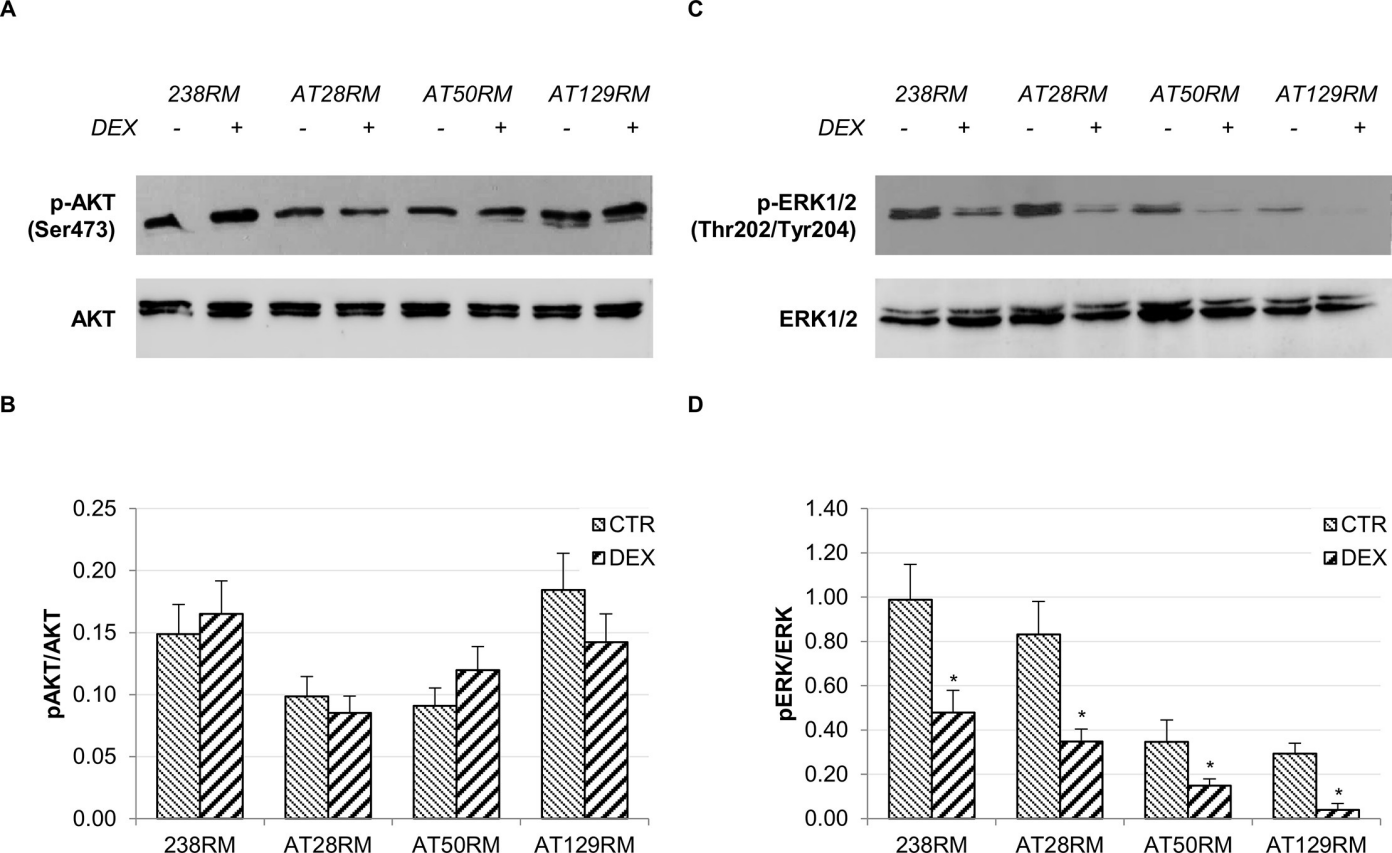
Effect of DEX on the PI3K/AKT pathway and ERK1/2 pathway. The activation status of the activating kinases AKT and ERK1/2 was investigated in WT and AT cells. **A),C)** Western blot analysis for phosphorylated AKT/total AKT and phosphorylated ERK1/2/total ERK1/2, respectively, in WT (238RM) and AT (AT28RM, AT50RM, AT129RM) cells treated with 100 nM DEX for 24 hours or not treated. **B),D)** p-AKT and p-ERK1/2 abundance was quantified and normalized, respectively, by the value of total AKT and total ERK1/2 signal. Blots shown are representative and values are the means and SEM of four independent experiments (Wilcoxon signed rand test; *two-tailed p-values<0.05).

We first investigated the “activating kinase” branch involving the AKT and ERK1/2 pathways (**Fig 1A**, third branch). Neither AKT nor ERK 1/2 were found to be activated after 24h treatment with DEX, thus excluding their involvement in NRF2 activation (**Fig 2**). Not only were ERK1/2 not activated, but we also found a significant reduction in the phosphorylation status. The de-phosphorylation observed in all cells (both wild type and AT) after DEX may be imputable to the short-term overexpression of several dual-specificity phosphatase genes (DUSP1-3-6-7-8-11-14-16-22) as reported in [24, 35]. This is in contrast with our previous report showing that AKT and ERK1/2 were partially activated by drug treatment [22]. However, it must be emphasized that this activation occurred only at longer drug exposure time (72h). Moreover, it was directly related to the induction of the “mini-ATM” protein isoform, which only occurred after 48h of DEX exposure. In any case, the findings of the present study suggest that DEX could exert its effects on the “inhibitory kinase” branch rather than via direct PI3K-activation.

### Effect of DEX on NRF2-inhibitory kinases

As mentioned in the introduction, in pathological conditions, the persistence of inflammatory or oxidative statuses can lead to the chronic activation of inhibitory kinases, such as GSK3 and/or p38 MAPK, which exert a negative regulation on the transcription factor NRF2 (**Fig 1A**, fourth branch). Accordingly, we hypothesized that DEX could have an indirect activating effect on NRF2 by blocking one or both of these inhibitory kinases.

#### Effect of DEX on the GSK3 inhibitory kinase

**Fig 3:**
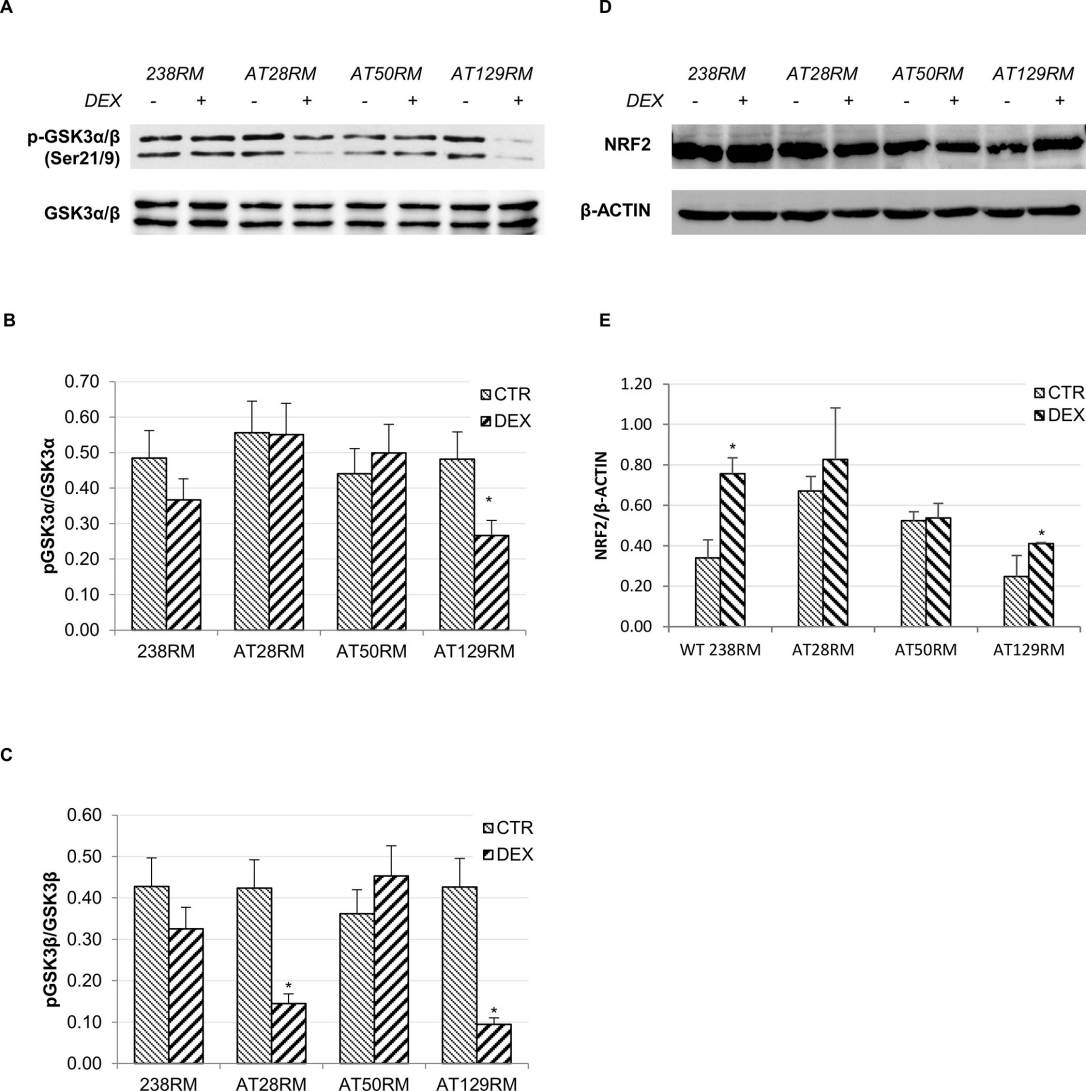
GSK3 is not inhibited by DEX in AT cells. The activation status of the inhibitory kinase GSK3 was investigated in WT and AT cells. **A)** Western blot analysis for phosphorylated GSK3-α/β and total GSK3-α/β, in WT (238RM) and AT (AT28RM, AT50RM, AT129RM) cells treated as reported in Fig 2**. B),C)** Quantification of p-GSK3-α/total GSK3-α and of p-GSK3-β/total GSK3-β ratio, respectively. **D)** Western blot analysis for total NRF2 protein levels in the four cell lines used in this study, treated as in Fig 2. β-ACTIN served as a loading control. **E)** Quantification of the relative amounts of NRF2 in the total cell extracts of WT and AT cells shown in D. Blots shown are representative and the histograms are the means and SEM of four independent experiments. (Wilcoxon signed rand test; *two-tailed p-values<0.05).

Evidence suggests that GSK3 is able to directly (or indirectly by Fyn) phosphorylate NRF2 in specific residues, leading to NRF2 degradation in a KEAP1-independent manner, in particular via the adaptor protein β-TrCP [31, 36]. Activated GSK3 is thought to phosphorylate NRF2 at specific serine residues (i.e. Ser342 and 347), but unfortunately, no phospho-specific antibodies are yet available to investigate this phenomenon. However, GSK3 activity is in turn regulated by phosphorylation on specific serine residues. In particular, phosphorylation in Ser21-GSK3α and Ser9-GSK3β acts as a switch-off mechanism for GSK3 activity since AKT is the main kinase phosphorylating GSK3 in the said positions. Hence, we investigated GSK3 phosphorylation in our samples (Fig 3A). Our results showed that the phosphorylation status of the GSK3α and β isoforms was decreased, respectively, in one out of three and two out of three AT cell lines after drug treatment (Fig 3B and 3C), which is consistent with a further activation of the kinase rather than the expected inactivation. However, the total NRF2 protein levels, determined by immunoblotting analysis on the same experimental samples used to study the GSK3 phosphorylation status, showed a statistically significant increase in the wild-type 238RM and AT129RM cell lines, while the NRF2 protein levels remained unchanged in the other two AT cell lines (Fig 3D and 3E). The absence of a correlation between GSK3 activation and NRF2 degradation appears to exclude the direct involvement of this kinase in the DEX-mediated NRF2 activation pathway. Nevertheless, the increased phosphorylation of GSK3 was rather unexpected considering the above reported lack of DEX-dependent activation of AKT in the AT cell lines. In agreement with our findings, proof of concept reports a “long-term” response (12h, [37]) that results in AKT down-regulation and consequent GSK3 activation in disease conditions, including chronic inflammation and neurologic diseases [38].

#### Double-sided effect of DEX on Fyn kinase

As shown in the fourth branch of Fig 1A, GSK3 is also thought to act via Fyn tyrosine kinase. Further evidence suggests that the GSK3 inhibitory effect is exerted on the regulation of the nuclear export of NRF2 by acting as upstream activator of Fyn [33]. According to Niture et al., NRF2 activation is a multi-phase process that involves: a “pre-induction phase”, during which negative regulators are exported out of the nucleus, followed by an “induction phase”, during which NRF2 is imported into the nucleus, and a “post-induction phase”, during which the negative regulators (e. g. KEAP1, Fyn, Bach1) are recruited into the nucleus to shut down the oxidant response [33]. Taken together, these considerations led us to investigate the effect of DEX on the Fyn negative regulator.

**Fig 4:**
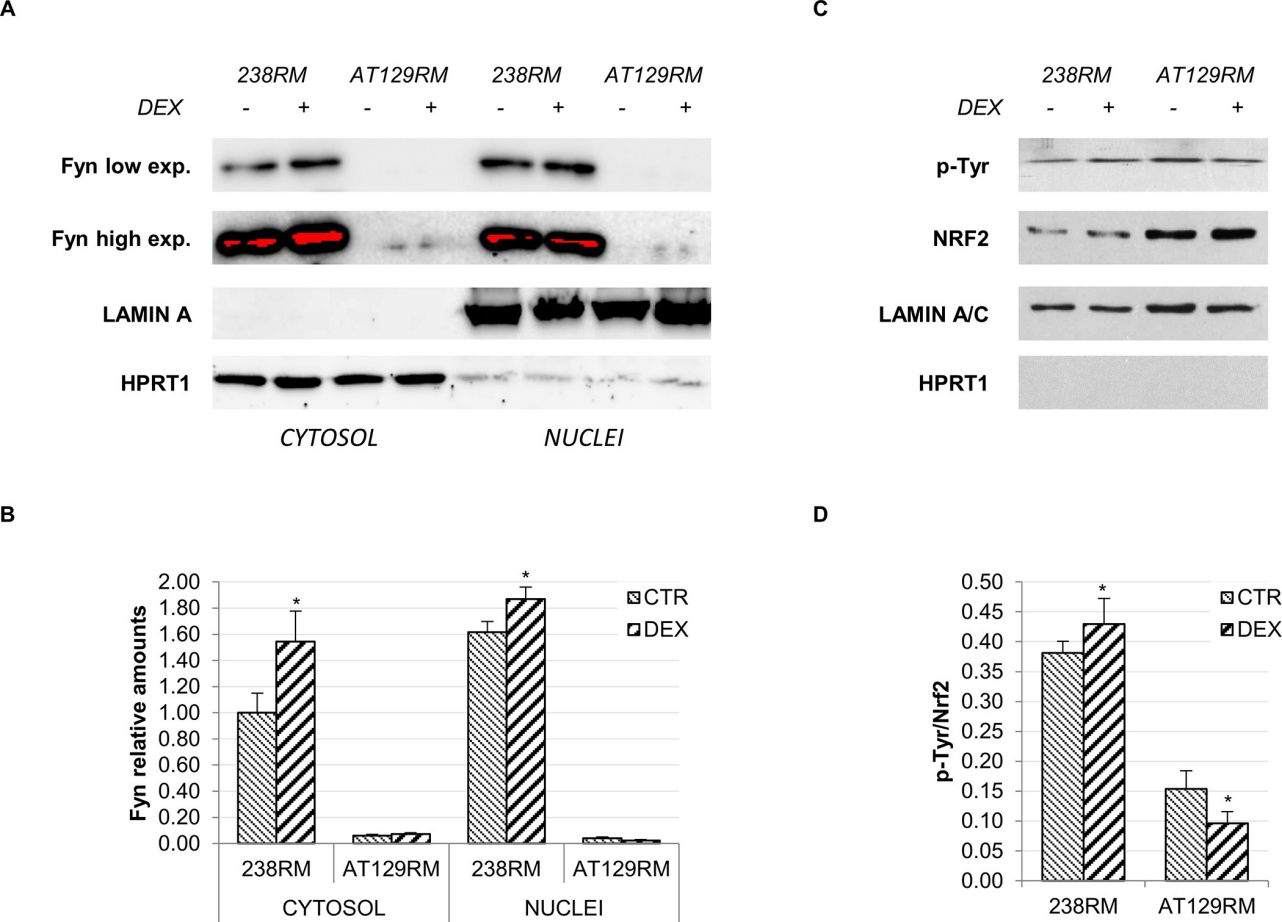
DEX activates a delayed response in WT cells but not in AT cells. Fyn nuclear import and NRF2 phosphorylation were detected in WT and AT cells. **A)** Western blot analysis for Fyn in the cytosolic/nuclear fractions of WT (238RM) and AT (AT28RM, AT50RM, AT129RM) cells treated as reported in Fig 2. HPRT1 and LAMIN A served as a loading control for the cytosolic/nuclear extracts, respectively. **B)** Quantification of the relative amount of Fyn in the cytosol and nuclei of WT and AT cells is shown. **C)** Western blot analysis for phosphorylated NRF2 and total NRF2 in the WT (238RM) and AT (AT129RM) cell lines, treated or not with DEX as above. After nuclei separation and lysis, NRF2 was immunoprecipitated and probed with either anti phospho-tyrosine or anti-NRF2. LAMIN A served as a loading control; HPRT1 was used to assess the lack of cross contamination and the consequent nuclear localization of NRF2. **D)** Quantification of the p-NRF2/NRF2 ratio. Blots shown are representative and the histograms are the means and SEM of four independent experiments (Wilcoxon signed rand test; *two-tailed p-values<0.05).

In particular, in the post-induction phase, activation of Fyn by phosphorylation, leads to the nuclear shift of Fyn itself. Once inside the nucleus, Fyn phosphorylates the tyrosine 568 of NRF2, resulting in the detachment of the transcription factor from target genes and subsequent nuclear export [39]. As mentioned above, this is a delayed response triggered to shut down NRF2 gene transcription when redox homeostasis is achieved, and, as reported in [40], it occurs by a two-step mechanism: detachment of NRF2 from ARE-dependent genes to stop transcription followed by nuclear export of NRF2 itself, which is also mediated by KEAP1. To gain more insight into this response, we first evaluated the nuclear shift of Fyn after DEX, in both WT and AT cells, and then the phosphorylation status of NRF2 after immunoprecipitation. Regarding Fyn kinase, it is very intriguing that in the investigated AT cell lines, protein levels were very low; hence, the signal was detectable only after very long exposures. It should be noted that Fyn levels are highly variable in different cells and usually poorly expressed in lymphoid cells [41, 42]. Indeed, we found low Fyn levels in several AT cell lines, while the WT showed higher Fyn levels (**S1 Fig**). To the best of our knowledge, no correlations exist between ATM and Fyn protein expression; however, this point certainly warrants further investigation. **Fig 4A** shows that, in AT cells, Fyn is only cytoplasmic, while in WT cells, where the Fyn content is much higher, a portion of protein is indeed shifted into the nucleus. This is consistent with the establishment of the above-mentioned delayed response and the shutdown of transcription in WT cells. It is noteworthy that in our previous work we observed that 24 hours after DEX treatment, while NRF2 was still nuclear in both, in WT cells it was not transcribing ARE genes, while in AT cells it was [25]. In support of this hypothesis, the phosphorylation status of immunoprecipitated NRF2 confirmed a higher rate of Tyr phosphorylation in WT cells after DEX. By contrast, in AT cells, we found a significant reduction in Tyr phosphorylation accordingly with the lack of nuclear Fyn (**Fig 4C and 4D**). This is consistent with the pre-induction phase in which unknown kinases phosphorylate Fyn to mediate its nuclear export and degradation [33].

However, even if the lack of Fyn recruitment in AT cells can explain the prolonged activation of NRF2 and the non-appearance of the post-induction phase, it does not account for the observed triggering of the “induction phase”. Therefore, we shifted our attention to the second inhibitory kinase, p38.

#### Effect of DEX on the p38 inhibitory kinase

The p38 subfamily of MAPKs regulates gene expression in response to various extracellular stimuli and exerts a critical role in inflammation and stress response [43]. P38 is activated by dual specificity MAP kinases that phosphorylate p38 on Thr180 and Tyr 182 [43]. Inhibition of MAPK by glucocorticoids, via induction of MAPK Phosphatase-1 (DUSP1), has been extensively documented [44, 45]. In addition, previous investigations have shown that ATM plays a crucial role in cell proliferation by suppressing ROS-p38 MAPK signalling and *ATM*^*(-/-)*^ cells show enhanced activity of the p38 mitogen-activated protein kinase (MAPK) [46]. In particular, Kim and Wong reported that treatment with antioxidants or with p38 MAPK inhibitors might restore normal proliferation. Taken together, these observations suggest that DEX could act as an NRF2 inducer by disrupting the negative regulation of p38.

**Fig 5:**
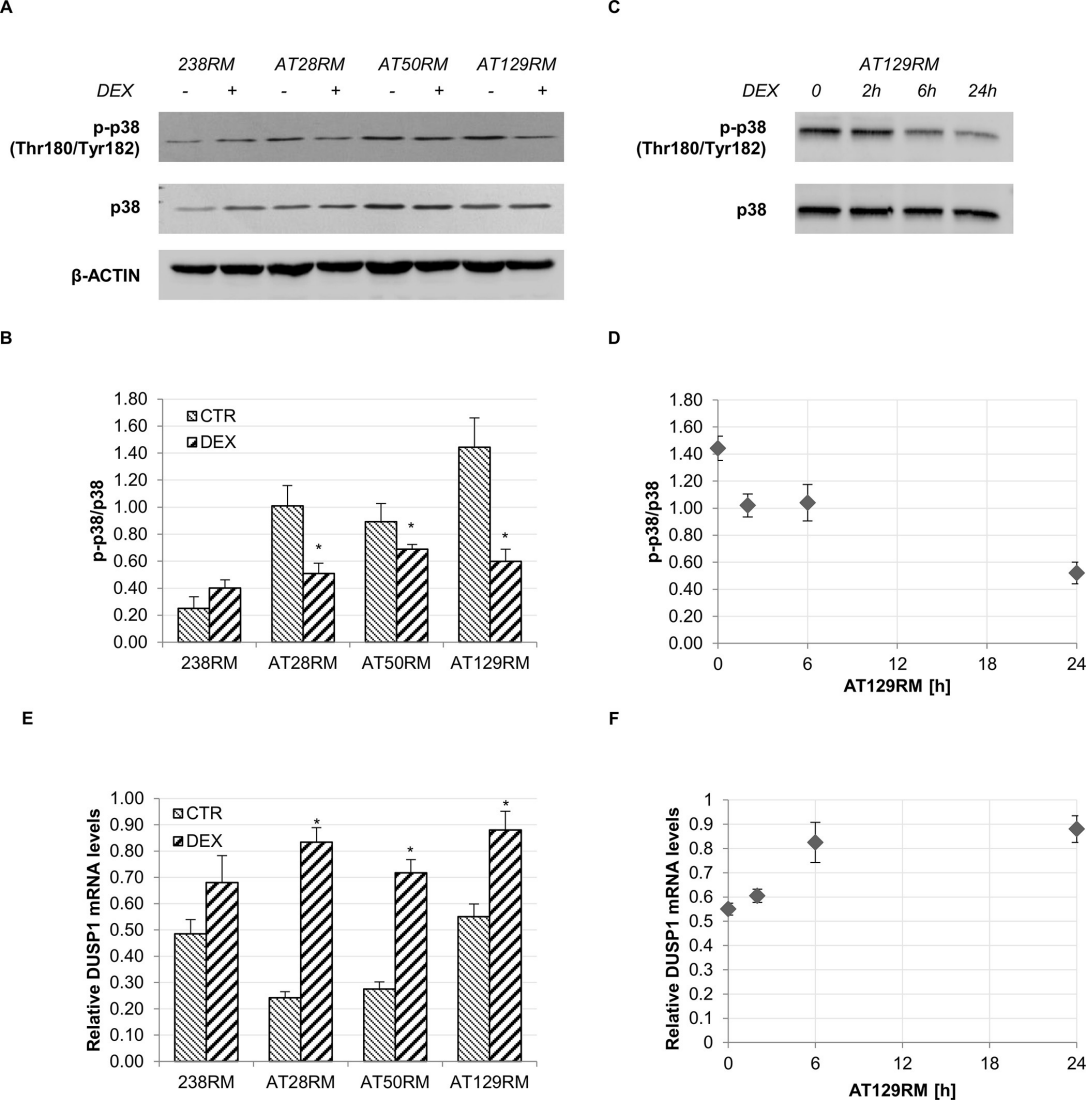
P38 switch-off by DEX and concomitant DUSP1 switch-on. **A)** Western blot analysis for phosphorylated p38 and total p38 in WT (238RM) and AT (AT28RM, AT50RM, AT129RM) cells treated as reported in Fig 2. β-ACTIN served as a loading control. **B)** Quantification of p-p38/total p38 ratio. **C)** Western blot analysis for phosphorylated p38 and total p38 in the AT129RM cell line treated with DEX for 2-6-24 h or not treated. **E)** Relative DUSP1 mRNA level in WT and AT cells treated as in A). **D),F)** Time-course of p38 switch-off and DUSP1/MKP1 switch-on, respectively, in the AT129RM cell line treated with DEX for 2-6-24h or not treated; asterisks refer to the respective t0 value. Blots shown are representative and the histograms (B,E) are the means and SEM of four independent experiments (Wilcoxon signed rand test; *two-tailed p-values<0.05).

To verify this hypothesis we evaluated the p38 activation status in WT and AT cells with or without DEX. In agreement with the findings of Kim and Wong [46], we observed that, at basal conditions, p38 is indeed more activated in AT cells than it is in WT cells (**Fig 5A and 5B**). In addition, we found that 24-hour DEX treatment was able to reduce this activation significantly. In addition, the time-dependent inhibition of p38 by DEX in AT129RM cells clearly suggests that the greatest effect is after 24 hours (**Fig 5C**).

Inactivation of MAPK in mammalian cells is achieved by a family of dual-specificity MAPK phosphatases (MKP), which target the two critical phosphorylation sites in the activation loop of MAPK. Among the characterized MKPs, MKP1 is known to be responsible for the de-phosphorylation and consequent inactivation of p38 [47, 48]. As reported above, it is well known that dexamethasone inhibits p38 MAPK by trans-activating the dual specificity phosphatase 1 gene (*DUSP1*), which codes for the corresponding MAPK phosphatase 1 (MKP1) protein [44, 45]. We therefore investigated whether p38 switch-off could be due to direct activation of MKP1, and we observed a significant transactivation of DUSP1 after DEX treatment **(Fig 5E)**, consistent with the previously observed p38 inhibition (**Fig 5B**). Panels D, F and G in **Fig 5** show the time-dependent inactivation of p38 accompanied by the concomitant DUSP1 mRNA induction and the corresponding MKP1 protein expression in the AT129RM cell line.

To verify whether NRF2 activation was to some extent attributable to the observed p38 inhibition by DEX, we investigated whether a p38 inhibitor could mimic the same effect of the glucocorticoid analogue on NRF2 activation, in the WT cell line and in one AT cell line as models.

**Fig 6:**
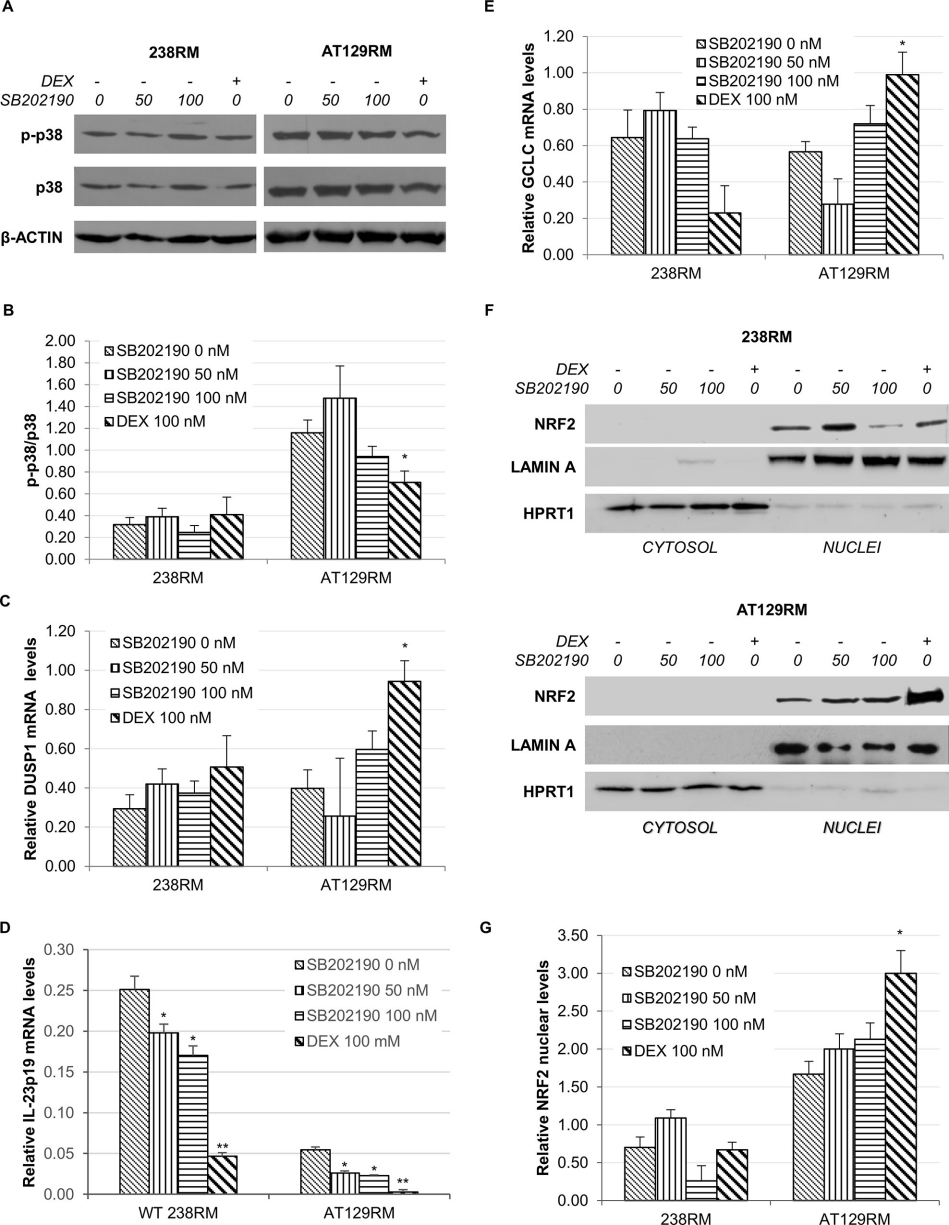
P38 inhibitor versus DEX effects on NRF2 activation. **A)** Western blot analysis for phosphorylated p38 and total p38 in WT (238RM) and AT (AT129RM) cells treated with increasing amounts of the p38 inhibitor SB202190 (0-50-100 nM) compared to DEX (100 nM). β-ACTIN served as a loading control. **B)** Quantification of p-p38/total p38 ratio. **C)** The histogram shows the relative DUSP1 mRNA level in WT and AT samples treated as in A. **D)** Relative IL-23p19 mRNA levels in WT (238RM) and AT (AT129RM) cells treated as in A. B2M mRNA was used as normalizer. **E)** GCLC (a selected NRF2 target gene) mRNA level in WT and AT129RM cells after both SB202190 and DEX treatment, as indicated. **F)** Western blot analysis for NRF2 in the cytosolic/nuclear fractions of WT and AT129RM cells treated as in A. HPRT1 and LAMIN A served as a loading control for the cytosolic/nuclear extracts, respectively. **G)** Quantification of the relative amount of NRF2 in the nuclei of WT and AT129RM cells treated as in A. Blots shown are representative and the values are the means and SEM of four independent experiments (Wilcoxon signed rand test; *two-tailed p-values<0.05 refers to DEX vs the respective control cell line at 0 nM SB202190).

To this end, we treated WT and AT129RM cells with increasing doses (50 and 100 nM) of the p38 inhibitor, SB202190. SB202190, which does not affect other MAKPs (i.e. JNK, ERK1/2, etc.), is a highly selective potent inhibitor of all p38 isoforms, with an IC50 of 50–100 nM [49]. In parallel, we administered the DEX treatment. In **Fig 6A and 6B** we can observe how DEX was able to significantly inactivate p38 only in the AT cell line, and this occurred in an MKP1-dependent manner, as supported by the concomitant DUSP1 mRNA induction (**Fig 6C**), while the SB202190 inhibitor was not effective in significantly lowering p-p38 levels in either cell model. Nevertheless, the SB202190 inhibitor acts in a different way than DEX (it binds to the active protein lowering its affinity for the ATP substrate, thus not affecting the phosphorylation status of the protein). We therefore used a different approach to demonstrate effective p38 inhibition. Considering that active p38 is able to favour the stability of some pro-inflammatory transcripts (such as IL-23p19) [50], we assessed whether SB202190 was indeed able to affect p38 activity by determining the IL-23p19 mRNA levels in our experimental conditions. **Fig 6D** shows that the inhibitor actually decreased the levels of the IL-23p19 transcript in a dose-dependent manner. After having verified the efficacy of both drugs in inhibiting p38, we compared the expression of a selected NRF2 target gene used as a control for transcription factor activation. We found that DEX was able to significantly increase the glutamate-cysteine ligase catalytic subunit (GCLC) mRNA, whereas SB202190 was not (**Fig 6E**).

In light of these results, we turned our attention to the nuclear shift of NRF2 demonstrated in [25] and repeated the experiments in the presence of the p38 inhibitor. We found that, after SB202190 treatment, NRF2 nuclear levels in AT cells are clearly lower than those of cells treated with DEX (**Fig 6F and 6G**); this is consistent with the low (not significant) induction of model target genes (**Fig 6E**). Taken together, these results led us to rule out the contribution of p38 in NRF2 activation after DEX treatment. We therefore refocused our attention on the first branch of our outline in **Fig 1A**, which addresses the nuclear-cytoplasmic shuttling of NRF2 under oxidative conditions.

### Effect of DEX on KEAP1 and the oxidative pathway

One of the most studied mechanisms of activation of NRF2 are the redox stressors, which put the Kelch-like ECH-associated protein 1 (KEAP1) out of play. KEAP1 is an NRF2 repressor that is inactivated by stress-stimulated modification of thiols elicited by oxidants and/or electrophiles [34]. Modifications of Cys residues in KEAP1 block cullin-3 ring ubiquitin ligase complex activity allowing newly synthesized NRF2 to accumulate and move to the nucleus (**Fig 1A and 1B**). KEAP1 is a relatively cysteine-rich protein that is mainly located in the cytosol as a dimer targeting NRF2 to proteasome degradation [51].

#### KEAP1 cytosolic-nuclear shuttling

**Fig 7:**
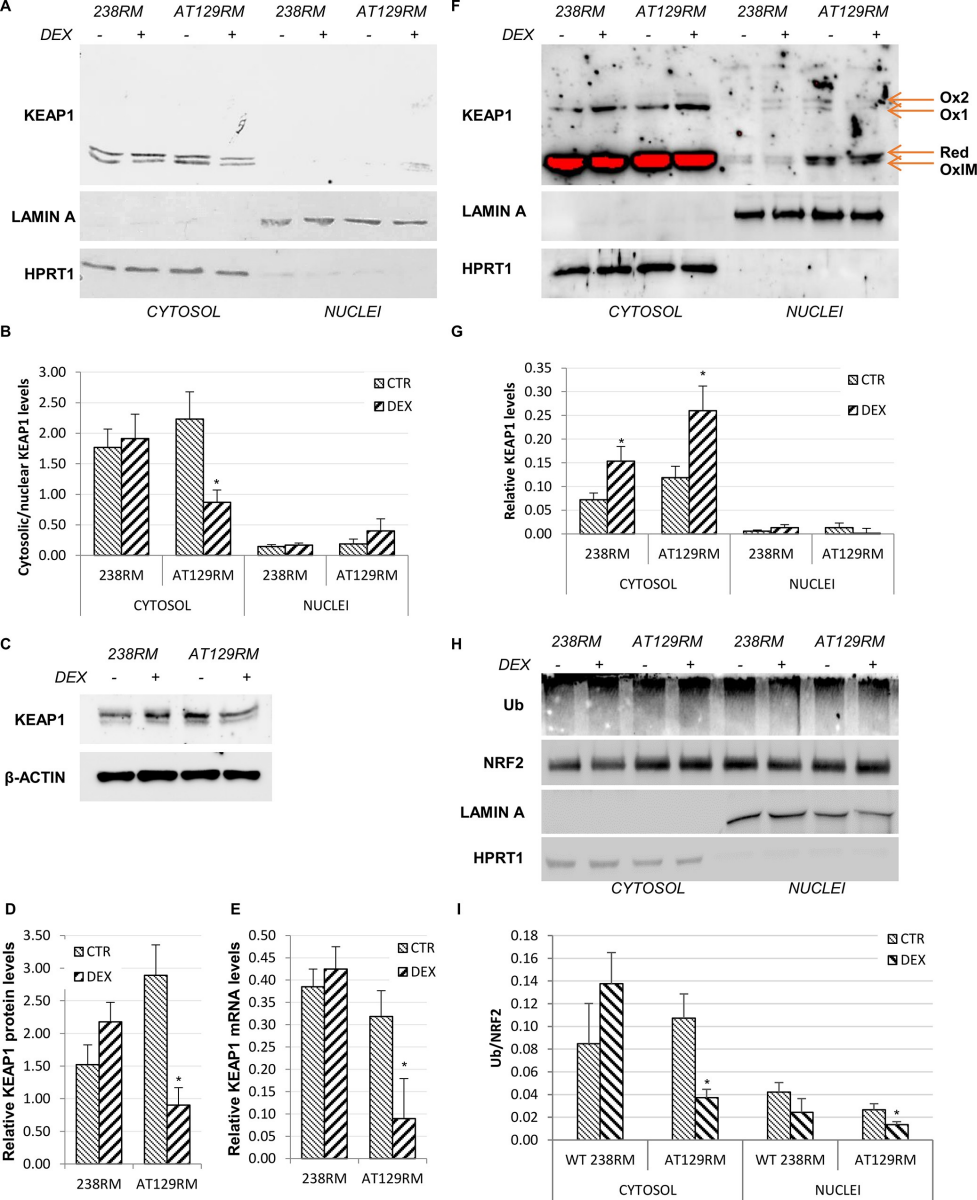
KEAP1 negative regulation and NRF2 ubiquitination status. **A)** Western blot analysis and **B)** quantification of abundance of KEAP1 in the cytosolic/nuclear fractions of WT (238RM) and AT (AT28RM, AT50RM, AT129RM) cells, treated with DEX or not treated. HPRT1 and LAMIN A served as a loading control for the cytosolic/nuclear extracts, respectively. **C)** Western blot analysis and **D)** quantification of total KEAP1 protein expression in total protein extracts in the WT cell line and in one AT cell line. β-ACTIN served as a loading control. **E)** KEAP1 mRNA level in the same cellular samples analysed in D. HPRT1 was used as the housekeeping gene for data normalization. **F)** Redox western KEAP1: nuclear and cytosolic extracts from WT and AT cells, treated or not with DEX, were analysed by SDS-PAGE under nonreducing conditions to highlight oxidized forms of KEAP1, and probed with anti-KEAP1 antibody. The arrows indicate the oxidized slow (Ox1 and Ox2), fast (OxIM) and reduced (Red) KEAP1 species. HPRT1 and LAMIN A served as a loading control for the cytosolic/nuclear extracts, respectively. **G)** Quantification of Ox1 KEAP1 abundance in the cytosolic/nuclear fractions, after normalization to the HPRT1 and LAMIN A signal, respectively. **H)** NRF2 ubiquitination in WT and AT cells, treated as in A. NRF2 protein was immunoprecipitated from both cytosolic and nuclear extracts and then immunoblotted with anti-NRF2 and anti-ubiquitin antibody. HPRT1 and LAMIN A served as a loading control for the cytosolic/nuclear extracts, respectively. **I)** Quantification of the Ub/NRF2 ratio. Blots shown are representative and the histograms are the means and SEM of four independent experiments (Wilcoxon signed rand test; *two-tailed p-values<0.05).

As we can observe in Fig 7A, under resting conditions, KEAP1 is mainly cytosolic in both WT and AT129RM cells, as we would expect. However, after DEX treatment, we found a significant reduction in cytosolic KEAP1 in AT cells, which is not compensated for by a comparable increase in the nuclear fraction (Fig 7B). Thus, we hypothesised that DEX could directly affect KEAP1 protein expression.

#### KEAP1 mRNA and protein expression

Indeed, we found a net decrease in the KEAP1 protein after DEX treatment, but only in AT cells (Fig 7C and 7D). To confirm our hypothesis, we tested whether the decreased protein level was paralleled by a downregulation of KEAP1 gene expression, and we found that KEAP1 mRNA was indeed significantly reduced after DEX treatment, only in AT cells (Fig 7E). The mRNA and protein expression levels were tested also in the other AT cell lines, confirming the downregulation (S2 Fig). This finding stands in contrast with what has been reported in the literature, according to which, the KEAP1 gene is up regulated by NRF2 itself as a negative feedback mechanism [52]. Nevertheless, considering that the marked effect was observed at the protein level, we wondered whether this reduction was also due to protein oxidation and subsequent degradation.

#### Redox Western KEAP1

Highly reactive cysteine residues, within the domains of KEAP1, set up at least three different redox sensor systems. Cys151 is crucial for oxidation by tBHQ and sulphoraphane, and can form a transient disulphide bridge with Cys151 in another KEAP1 subunit [32]; Cys273 and 288 recognize alkenals and prostaglandins [32, 53–55], while Cys226 and 613 recognize metals and form transient intramolecular disulphide bridge [32, 53]. Hence, as a final line of inquiry, we sought to determine whether DEX was able to affect the oxidation status of KEAP1 by redox western blot as reported in [32]. Hence, we performed electrophoretic runs on the same cytosolic/nuclear extracts under both non-reducing and reducing SDS-PAGE. Our results in **Fig 7F** show that oxidized forms of KEAP1 (namely Ox1 and Ox2) are indeed detectable in non-reducing SDS-PAGE. These two higher isoforms are supposed to be formed by an intermolecular disulphide bridge between the cysteine residues of two KEAP1 molecules (homodimer) or KEAP1 together with another protein (heterodimer) [32]. However, as reported by Fouquet et al., in both cases, KEAP1 homo/hetero-dimer is no longer able to interact with NRF2 to keep it in the cytosol. In particular, their model suggests that the stress signal and/or electrophiles disrupt the KEAP1-Cul3 axis by modifying the Cys151 residue on KEAP1, thus stopping the inhibitory effect and leading to NRF2 release. Quantifying the Ox1 bands in our blots **(Fig 7G)**, we found an increase in WT and especially in AT cells after DEX treatment, which is consistent with a further inactivation of KEAP1 and consequent activation of NRF2. To directly prove that KEAP1 is indeed responsible for modulating NRF2 after DEX treatment, we investigated whether the increase in the oxidized form of KEAP1 corresponds to alterations in NRF2 ubiquitination levels. To this end, NRF2 protein was immunoprecipitated from both cytosolic and nuclear extracts and then immunoblotted with anti-ubiquitin antibody. The results obtained show that NRF2 ubiquitination status was indeed decreased after DEX, which is consistent with the increased oxidation of KEAP1 (**Fig 7H and 7I**). To the best of our knowledge, this is the first report addressing the effect of DEX as an NRF2 inducer that acts by disrupting the NRF2-KEAP1 complex. Other authors [56–58] have described a similar effect, but it must be emphasized that they used a Dexamethasone that was modified with a mesylate group, which is responsible for the oxidative properties.

## Conclusions

In conclusion, in the present investigation we have shown that in AT cells, the activation of NRF2 by the glucocorticoid analogue DEX does not occur via activating or inactivating kinases, as commonly described in the literature. In fact, neither AKT nor ERK1/2 activating kinases were activated and GSK3 inactivating kinase was not inhibited, thus excluding their involvement in NRF2 activation. Regarding the effect of DEX on the p38 MAPK inactivating kinase, despite a significant p38 inhibition, this effect does not seem to have a role in NRF2 activation or stabilization. Our results suggest that the effect of DEX is more likely exerted directly on the KEAP1 inhibitory protein and that this effect involves the oxidative pathway. As we have shown, both KEAP1 mRNA and protein levels are reduced after drug treatment. In addition, an increase in oxidized and inactive forms of KEAP1, which corresponds to a decrease in NRF2 ubiquitination status, was observed. This result is consistent with a recent study describing the transcriptomic and proteomic profile of AT cells treated with DEX. The study described the upregulation of several proteins involved in protein metabolism and processing in the endoplasmic reticulum, protein folding and post-translational modifications [27]. Taken together, our findings suggest that NRF2 induction upon treatment with DEX is not mediated by p38, but rather, by KEAP1 inhibition and that is sustained by a delay in the post-induction phase, via Fyn kinase, which keeps NRF2 active in the nucleus. However, further investigations are necessary to gain a better understanding of the long-term effects of DEX in this phase and in particular, the regulation of Fyn expression in AT cells.

## Supporting information

S1 Fig Fyn protein expression in WT and AT cell lines. **A),B)** Western blot analysis for Fyn in the total extracts of WT (238RM) and AT (AT28RM, AT50RM, AT129RM) cells treated with DEX or not treated, as indicated. β-ACTIN served as a loading control. **C), D)** Quantification of the relative amount of Fyn protein in the total cell extracts of WT and AT cell lines tested in A and B. Blots shown are representative and the histograms are the means and SEM of three independent experiments.
(DOCX)

S2 Fig KEAP1 mRNA and protein expression in AT cell lines. **A)** KEAP1 mRNA level in AT28RM and AT50RM cells treated with DEX or not treated, as indicated. HPRT1 was used as the housekeeping gene for data normalization. **B)** Western blot analysis for KEAP1 in the total extracts derived from the same cell lines analysed in A. β-ACTIN served as a loading control. **C)** Quantification of the relative amount of KEAP1 protein in the total cell extracts of AT cell lines tested in B. Blots shown are representative and the histograms are the means and SEM of four independent experiments (Wilcoxon signed rand test; *two-tailed p-values<0.05).
(DOCX)

## Abbreviations

AT: Ataxia Telangiectasia
DEX: Dexamethasone
DMSO: Dimethylsulfoxide
DUSP1: Dual Specificity Phosphatase 1
GCLC: Glutamate-Cysteine Ligase Catalytic Subunit
KEAP1: Kelch-like ECH-associated protein 1
LCLs: EBV-transformed Lymphoblastoid B Cell Lines
NRF2: Nuclear factor erythroid 2-related factor 2
