# Supplementary material for: Activation of NRF2 by dexamethasone in ataxia telangiectasia cells involves KEAP1 inhibition but not the inhibition of p38
Source: PLoS One. 2019 May 20;14(5):e0216668. doi: 10.1371/journal.pone.0216668 (PMC6527213; doi:10.1371/journal.pone.0216668)
Supplement: S1 Fig — A),B) Western blot analysis for Fyn in the total extracts of WT (238RM) and AT (AT28RM, AT50RM, AT129RM) cells treated with DEX or not treated, as indicated. β-ACTIN served as a loading control. C), D) Quantification of the relative amount of Fyn protein in the total cell extracts of WT and AT cell lines tested in A and B. Blots shown are representative and the histograms are the means and SEM of three independent experiments. (DOCX) [file pone.0216668.s001.docx]

# **SUPPLEMENTARY FIGURE 1**


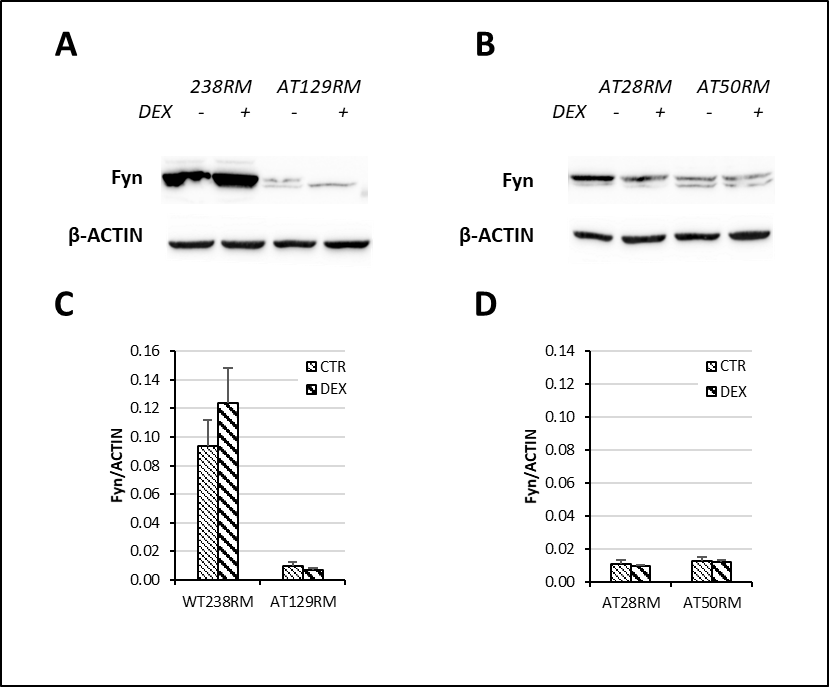


Figure S1 – Fyn protein expression in WT and AT cell lines. A),B) Western blot analysis for Fyn in the total extracts of WT (238RM) and AT (AT28RM, AT50RM, AT129RM) cells treated with DEX or not treated , as indicated. β-ACTIN served as a loading control. C), D) Quantification of the relative amount of Fyn protein in the total cell extracts of WT and AT cell lines tested in A and B. Blots shown are representative and the histograms are the means and SEM of three independent experiments.
