# Supplementary material for: Activation of NRF2 by dexamethasone in ataxia telangiectasia cells involves KEAP1 inhibition but not the inhibition of p38
Source: PLoS One. 2019 May 20;14(5):e0216668. doi: 10.1371/journal.pone.0216668 (PMC6527213; doi:10.1371/journal.pone.0216668)
Supplement: S2 Fig — A) KEAP1 mRNA level in AT28RM and AT50RM cells treated with DEX or not treated, as indicated. HPRT1 was used as the housekeeping gene for data normalization. B) Western blot analysis for KEAP1 in the total extracts derived from the same cell lines analysed in A. β-ACTIN served as a loading control. C) Quantification of the relative amount of KEAP1 protein in the total cell extracts of AT cell lines tested in B. Blots shown are representative and the histograms are the means and SEM of four independent experiments (Wilcoxon signed rand test; *two-tailed p-values<0.05). (DOCX) [file pone.0216668.s002.docx]

## **SUPPLEMENTARY FIGURE 2**


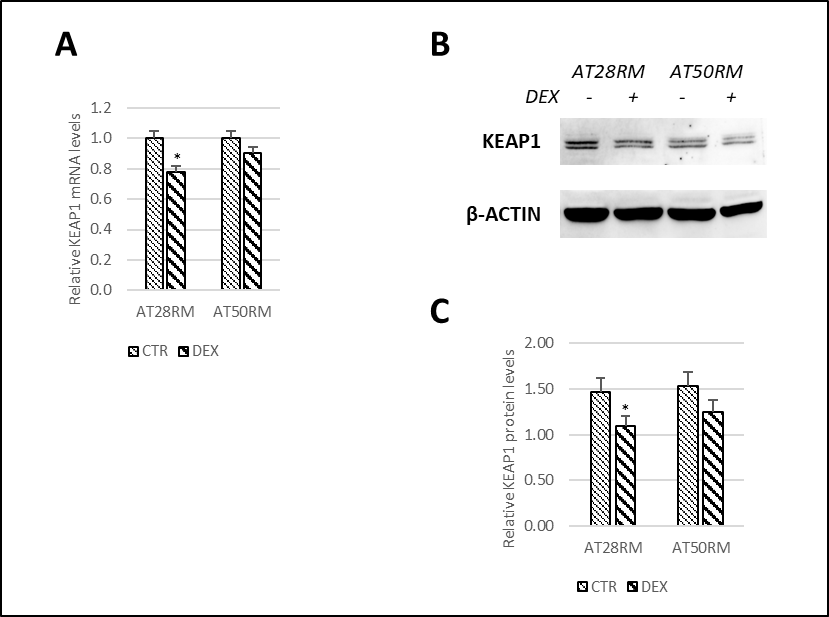


Figure S2 – KEAP1 mRNA and protein expression in AT cell lines. A) KEAP1 mRNA level in AT28RM and AT50RM cells treated with DEX or not treated, as indicated. HPRT1 was used as the housekeeping gene for data normalization. B) Western blot analysis for KEAP1 in the total extracts derived from the same cell lines analysed in A. β-ACTIN served as a loading control. C) Quantification of the relative amount of KEAP1 protein in the total cell extracts of AT cell lines tested in B. Blots shown are representative and the histograms are the means and SEM of four independent experiments (Wilcoxon signed rand test; *two-tailed p-values<0.05).
